# Clinically defining the opioid-exposed birthing person and infant as a dyad to support bedside care, surveillance, and research

**DOI:** 10.3389/fped.2024.1349102

**Published:** 2024-05-07

**Authors:** Shahla M. Jilani, Jonathan M. Davis, David Goldstein, Matthew Grossman, Lauren M. Jansson, Mishka Terplan, Hendrée E. Jones

**Affiliations:** ^1^ Office of the Assistant Secretary for Health, U.S. Department of Health and Human Services, Washington, DC, United States; ^2^ Division of Newborn Medicine, Tufts Medical Center, Boston, MA, United States; ^3^ The Tufts Clinical and Translational Science Institute, Boston, MA, United States; ^4^ Department of Pediatrics, Yale School of Medicine, New Haven, CT, United States; ^5^ Department of Pediatrics, Johns Hopkins University School of Medicine, Baltimore, MD, United States; ^6^ Center for Addiction and Pregnancy, Baltimore, MD, United States; ^7^ Friends Research Institute, Baltimore, MD, United States; ^8^ Department of Obstetrics and Gynecology, School of Medicine, The University of North Carolina at Chapel Hill, Chapel Hill, NC, United States; ^9^ Department of Psychiatry & Behavioral Sciences, Johns Hopkins University School of Medicine, Baltimore, MD, United States

**Keywords:** opioids, neonatal abstinence syndrome, withdrawal, dyad, outcomes

## Abstract

**Introduction:**

An increased incidence of maternal opioid use disorder (OUD) and neonatal abstinence syndrome (NAS) has prompted recommendations supporting a dyadic approach to care for birthing persons and their infants. However, there are no consensus guidelines outlining how the dyad is clinically defined.

**Methods:**

To examine how the opioid-exposed birthing person-infant dyad has been defined for purposes of data collection and research, a literature review applying the RAND/UCLA Appropriateness Method was conducted.

**Results:**

The search yielded 320 abstracts, with 110 articles identified as having a dyadic focus. While no articles included a specific definition for the dyad, 33 (30%) contained a descriptive reference to the birthing person-infant dyad. Thematic analysis revealed eight recurring elements characteristic of the dyad: (1) engagement, (2) communication, (3) bonding, (4) attachment, (5) mutual responsiveness, (6) reciprocity, (7) synchrony, and (8) attunement. Integrating these elements revealed the interactional relationship between the opioid-exposed birthing person and infant as the foundational principle that defines the dyad.

**Discussion:**

This definition shifts the focus of the opioid-exposed dyad from two individual patient populations to an interactional relationship that has broad applicability for clinical use, public health data collection, and research considerations.

## Introduction

The U.S. opioid crisis has resulted in a widespread public health problem affecting many populations, including birthing persons and their infants. The incidence of maternal opioid use disorder (OUD) and neonatal abstinence syndrome (NAS; signs of withdrawal following *in utero* exposure to opioids and other substances) increased by 4.6 and 3.3 per 1,000 birth hospitalizations, respectively, from 2010 to 2017 (1). Although the well-being of birthing persons is essential to the health and development of their infants, many barriers exist to care for pregnant and postpartum persons with OUD. This includes a lack of support/care for the child and access to medications for OUD (MOUD) despite evidence supporting their use in pregnancy (2–4). Such access is important as previous findings have shown that while MOUD can increase the severity of NAS, they reduce overdose death and preterm birth (5, 6). While the interdependence of birthing persons and their infants are recognized, often care is structured such that parents and children are treated as individual and disconnected entities. Such action can lead to higher health care costs, poorer outcomes for parents and children, and missed opportunities for prevention and early intervention.

In view of addressing these care gaps, there is growing support for a dyadic-centered approach to care for substance-exposed birthing persons and infants (7–10). Given the inextricable link between birth persons and their infants, such an integrated approach, considering the needs of both, offers an essential strategy for clinical, surveillance, and research considerations. The definition of a dyad and the study of dyadic relationships has received decades of attention in the developmental literature. DW Winnicott (11) maintained that there is “no such thing as a baby; a baby is a part of a dyadic unit”. Attachment theory (Bowlby) (12) stressed the psychological interplay between parent and child and attunement (Stern) (13) emphasized the intersubjective relationship between the dyad's components. Yet when one aspect of the dyad struggles with a substance use disorder, mental health concerns, or any condition that affects the ability to function as a connected caregiver, these theoretical paradigms become skewed. Therefore, the operationalizing of this work at the bedside for providers from different fields of medicine and in policy research is of paramount importance. However, this has received little attention. Evidence-based criteria and/or consensus guidelines remain limited not only for the clinician, but also across surveillance and research pathways. Thus, what constitutes optimal dyadic care can vary significantly among providers, public health professionals, and researchers. More fundamentally, the concept of the dyad itself can vary in definition, breadth, and scope well beyond the birth relationship (i.e., parent-infant; caregiver-child, any two-person group)—prompting a need for foundational guidance that establishes how the dyad is defined, especially at the clinical bedside. Accordingly, the purpose of this study was to examine how the opioid-exposed birthing person-infant dyad has been defined in clinical research with an overarching goal of informing the development of a clinical definition that may be applicable to public health data collection and research considerations.

The current study applied the RAND/University of California Los Angeles (UCLA) Appropriateness Method (RAM) approach to the literature review. (14), This approach has been applied in numerous studies, particularly for questions with similar limitations in evidence-based criteria guiding practice (10, 15). Distinct from statistical methods used in meta-analyses, a RAM literature review enables integration of expert input with synthesis of existing literature to address key knowledge gaps (14). Accordingly, this RAM literature review was conducted by a Federal Steering Committee (FSC; a convened group of staff representing multiple federal agencies) together with an Expert Panel (EP) assembled to examine available literature with the following objectives: (1) summarize how the birthing person-infant dyad has been defined/described across the clinical research spectrum with a focus on exposure to opioids with/without other psychotropic substances, (2) synthesize knowledge gaps in definitional/descriptive criteria, and (3) identify key elements that may address definitional gaps. Together, these findings were used to derive a recommended clinical definition for the opioid-exposed birthing person-infant dyad that can be applied in the surveillance and research settings serving this population.

## Methods

### Assembly of the federal steering committee and expert panel

Conducted between January–June 2023, the initial step of this RAM literature review entailed assembly of the FSC and EP. The FSC was comprised of policy experts in maternal-child health across the following agencies: Agency for Healthcare Research and Quality, Office of the Assistant Secretary for Planning and Evaluation, Centers for Disease Control and Prevention, Health Resources and Services Administration, National Institute on Drug Abuse, Office of the Assistant Secretary for Health, National Institutes of Health Office of Research on Women's Health, and the Substance Abuse and Mental Health Services Administration. The five-membered EP was comprised of clinical and research experts in maternal-child health including addiction medicine, developmental pediatrics, hospital pediatrics, neonatology, and obstetrics and gynecology. Alongside the FSC and EP, a research team was assembled to coordinate and conduct the iterative review process. An internal fidelity process was designed for the abstract and subsequent full text reviews to optimize integrity and minimize inter-reviewer inconsistency. This process was an iterative 2-stage review by members of the research team assessing for agreement, disagreement, and need for further review. The overarching search strategy focused on clinical research that discussed the dyad in the context of *in utero* opioid exposure with/without other psychotropic substances.

### Application of RAND/UCLA appropriateness modified delphi method

As a framework for this study, the RAM literature review was designed to identify literature defining or describing the dyad across clinical research on birthing persons and infants with opioid with/without other psychotropic substance exposure. Synthesized literature underwent thematic/inductive analysis to delineate and/or identify themes definitional to and/or descriptive for the opioid exposed dyad. Identified themes were integrated to derive a clinical definition for the dyad.

### Keyword search

With the scope of the research targeted to *in utero* opioid exposures, keywords were tailored to reflect a birthing person-infant relationship. The EP generated a keyword search to capture the range of terms that have been used to describe the dyad as follows: mother-infant dyad, mother-fetus dyad, mother-child dyad, mother-baby dyad, mother-offspring dyad, parent-infant dyad, parent-fetus dyad, mother-infant pair, mother-fetus pair, mother-child pair, mother-baby pair, mother-offspring pair, parent-infant pair, parent-fetus pair. This was combined with terminology that specified a broad spectrum of opioid exposure including: opioid, opiate, narcotics, narcotic, drug, chemical, substance, analgesics, opioid and addict, dependent, abuse, habit, overdose, misuse, use disorder, opioid-related disorders, morphine dependence, heroin dependence, substance abuse, intravenous, oral, narcotic-related disorders, substance-related disorders, opium dependence.

Though polysubstance was a component of the primary research question, a comprehensive search including numerous (discrete) psychotropic substances was beyond the scope of this focused review. However, given the breadth of evidence supporting causal links between the prominent role of opioid exposure and neonatal withdrawal, the term opioid was explicitly included as a specific requirement. Other non-opioid and polysubstance exposures were more broadly denoted as substance, drug, or chemical exposures.

The search was run in PubMed, Web of Science, Scopus, PsycINFO, and Sociological Abstracts (no date limitations). An environmental scan using this keyword combination conducted in January 2023 yielded 294 abstracts with specificity to the primary research question—identifying criteria used to define the birthing person-infant dyad. In March 2023, as a measure to ensure no abstracts were missed in the environmental scan, one additional keyword “maternal” was added to the formal search and yielded 26 additional abstracts for review.

### Inclusion and exclusion criteria

To identify clinical studies which discuss a definition or description for the dyad, inclusion criteria were case controls, cohort trials, and randomized control trials; review articles, statement/guidance documents, and commentaries were also included to inform the discussion. Together, inclusion criteria targeted peer-reviewed publications with a focus on the dyad and/or dyadic health outcomes.

While using the terms “mother-child dyad” and “mother-child pair” in the keyword search was designed to optimize capture of abstracts relevant to the research question, it also yielded abstracts on dyads without a specific focus on *in utero* opioid exposure. As the research question focused distinctly on the dyad with *in utero* opioid with/without other substance exposures, retrieved abstracts discussing exposures beyond the prenatal period were removed. However, if an abstract discussed prenatal exposures longitudinally followed from infancy into childhood, it was included in the review.

**Figure 1:**
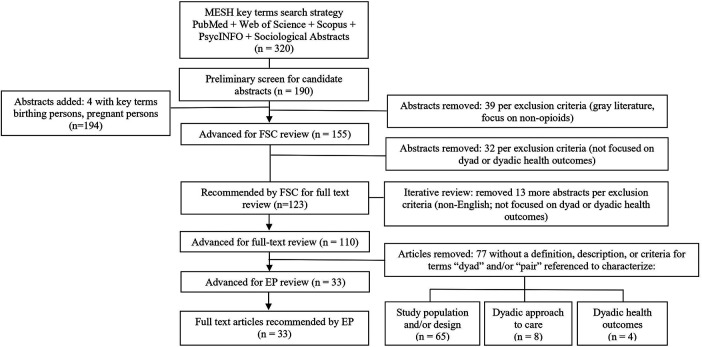
Preliminary screen of abstracts (*n* = 320) yielded 190 abstracts. Four abstracts were added inclusive of current terminology, and candidate abstracts (*n* = 155) were advanced for federal steering committee (FSC) review. Of 110 advanced for full-text review, 77 were removed per exclusion criteria; 33 were advanced for Expert Panel (EP) recommendations. MESH, medical subject headings.

The full set of abstracts (*n* = 320) underwent a preliminary screen to remove animal studies, duplicates, individual case reports, abstracts focused on birthing persons or infants (not on the dyad), and those without topical relevance yielding 190 candidate abstracts (Figure 1). The preliminary screen was not designed to serve as an in-depth review, but instead to determine whether the broad content of each abstract was within scope. As a measure to optimize inclusion of more recently applied terminology in the field, two new keywords were added in March 2023: birthing people/person and pregnant people/person with 4 additional abstracts added for review. From the preliminary screen, further exclusion was made of gray literature (dissertations, theses, other academic papers not peer-reviewed or published by traditional commercial publishers) and abstracts that did not include opioid exposure. Accordingly, candidate abstracts (*n* = 155) were advanced for abstract review by the FSC.

### Abstract review

The FSC conducted in-depth reviews of the 155 candidate abstracts, with 123 meeting inclusion criteria as dyad/dyadic focused and 32 excluded as not focused on the dyad (e.g., focused instead on either the birthing person or infant alone) or dyadic health outcomes (e.g., focused instead on laboratory biomarkers, toxicology, or other diagnostics) (Figure 1). Abstracts recommended for inclusion and exclusion by the FSC were independently examined by a single reviewer on the research team, applying the internal fidelity process. This resulted in agreement on 116 abstracts to advance for full-text review and disagreement on seven abstracts. For abstracts in question, an independent review was conducted by an additional researcher which determined that these articles focused on either birthing person or infant/child (not the dyad) and were removed. Of full-text articles, five were found to be non-English and one was no longer available from the publisher. In total, 110 abstracts with a focus on the birthing person-infant dyad were advanced for full-text review.

### Full-text review

A full-text review was conducted with a focus on identification of a definition, description, and/or criteria referencing the term dyad (Figure 1). A structured 2-step process was developed to conduct reviewer assessment: (1) specifying the terms: dyad(s), dyadic used interchangeably with pair(s) and (2) examination of each occurrence of this terminology to determine whether reference to a definition, description, and/or criteria was made in the article. The review was conducted per the established internal fidelity process with two expert clinician-researchers independently reviewing each full-text article (*n* = 110), assessing for agreement, disagreement, and need for further review. This internal fidelity review resulted in agreement on all elements identified as descriptive for the dyad by thematic analysis. Inter-reviewer agreement was achieved on exclusion of 77 articles which were found not to include a definition, description, and/or criteria for the dyad and were not advanced further. The remaining 33 were found to include a description for the dyad and were advanced for further analysis.

### Data analysis

To facilitate a structured review, articles advanced for analysis were first categorized based on emerging topical areas of focus: (1) types of birthing person-infant support, (2) presence or types of opioid/substance exposure and care recommendations, and (3) maternal psychiatric illness and opioid use. Across these categories, articles were then subcategorized by publication year and summarized by study focus and descriptive reference to the dyad. Each article was reviewed for dyad focused content and to collect data related to a description for the dyad. In the absence of a definition, a referenced description explaining what constitutes the dyad, its components, or characteristics was included in the analysis. Accordingly, detailed citations were abstracted from each article to construct a compilation of descriptive references for the dyad. Two members of the EP independently reviewed the compilation of cited descriptions and achieved agreement on inclusion of full-text articles and corresponding citations.

A thematic analysis of the identified descriptions was conducted using an inductive approach to delineate recurring themes (16). This analysis concentrated on identifying themes or descriptive elements distinct to the dyad, linked to the data abstracted from the articles. Themes emerged and were classified based on definitions derived from extant literature and provided below. Per the internal fidelity process, two members of the EP independently reviewed the thematic analysis and agreement was achieved on all elements identified as descriptive for the dyad.

## Results

Despite an emphasis on the dyad, 70% (77/110) of articles identified did not include a definition, description, or criteria for the dyad. While no definition was given, the terms dyad and pair were most often referenced to discuss the study population and/or design (e.g., inclusion/exclusion criteria, methodological design) (*n* = 65) (Figure 1). The other articles that met exclusion criteria discussed a dyadic approach to care for opioid-exposed birthing persons and their infants (*n* = 8) and dyadic outcomes (*n* = 4) without defining the term dyad.

The remaining 30% (33/110) of articles with a focus on the dyad included a description for the dyad beyond its use to reference the birthing person and infant. A structured full-text review of these articles revealed 3 topical categories focused on dyadic outcomes: (1) types of birthing person-infant support, (2) presence or types of opioid/substance exposure and care recommendations, and (3) maternal psychiatric illness and opioid/substance use. For topic group 1, 21 articles focused on MOUD, mental health therapies including psychotherapy, outpatient or residential substance treatment program, parent-infant interactions, and NAS management or other dyadic assessment/intervention programs (data not shown). For topic group 2, 7 articles focused on MOUD, mental health risk factors, family and legal considerations, parent-infant interactions, clinical management of NAS, and the effects of breastfeeding. For topic group 3, 5 articles focused on examination of parent-infant interactions and psychosocial risk factors.

Across these topical groups, a range of literature focusing on the opioid-exposed dyad was noted spanning 1982–2022 with gaps between publication years (without date filters applied in the keyword search), indicating a limitation in the availability of literature (Supplementary Table S1). A compilation of data was constructed with descriptive references to the dyad and thematically analyzed. Authors then identified key recurring elements as descriptive for the dyad. Descriptive elements identified were: (1) engagement; time spent participating in an interactive partnership, (2) communication; the conscious and subconscious interactions between a birthing person and infant, (3) bonding; feelings and emotions from the birthing person toward the infant, (4) attachment; early relationship development—the emotional connection and bond formed between birthing person and infant that provides the foundation for the infant’s emotional development, (5) mutual responsiveness; both birthing person and infant's influence on each other's emotions and behaviors as they grow together, (6) reciprocity; reciprocal social interactions between birthing person and infant, (7) synchrony; shared interest and behaviors that promote a mutually rewarding interaction, (8) attunement; the sharing of emotional experiences during interactions between birthing person and infant; empathy (12, 17–27).

**Figure 2:**
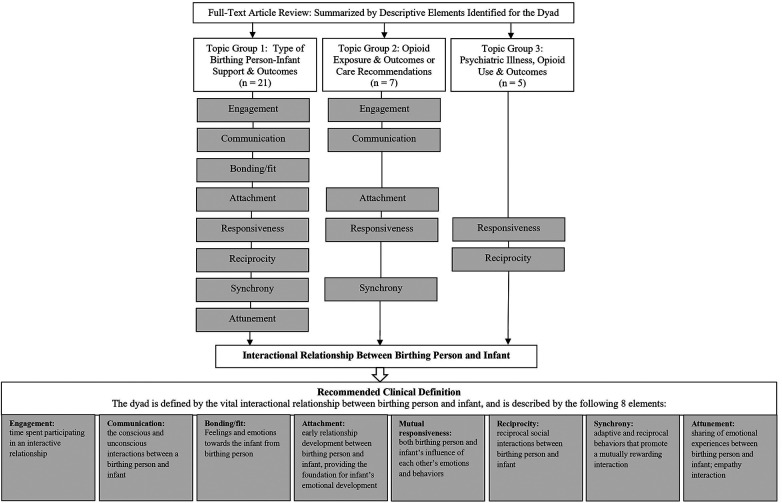
Thematic analysis of dyadic-focused articles categorized by topic area demonstrated eight recurring themes of elements describing the interactional relationship of the dyad as the foundational principle: (1) engagement, (2) communication, (3) bonding, (4) attachment, (5) responsiveness, (6) reciprocity, (7) synchrony, and (8) attunement, all represented in topic group 1 and subsets in groups 2 and 3; integrated to develop a recommended clinical definition.

Topic group 1 articles highlighted all descriptive elements outlined; group 2 articles included engagement, communication, attachment, responsiveness, and synchrony; group 3 articles included responsiveness and reciprocity (Figure 2). Individually, each element describes distinct aspects of the birthing person-infant dynamic from birth and spans the breadth of infant development; each element presents concepts identified in the literature as necessary for an understanding of characteristics essential to the dyad. Synthesis of these identified elements revealed one concept integral to the opioid-exposed dyad: the interactional relationship between birthing person and infant as the foundational concept that ties all eight elements together. Together, these findings were applied to delineate the relationship between the opioid-exposed birthing person and infant as definitional for the dyad, with eight characteristics descriptive of this unique relationship (Figure 2, Table 1).

**Table 1:** Examples of dyadic themes in the eight descriptive elements derived from depictions of the birthing person-infant dyad as described in the full text articles.

(1) **Engagement**
“Sensitivity to infant signals is considered as a dyadic construct; as any pattern of adult behavior that pleases the infant and increases the infant's comfort and attentiveness and reduces its distress and disengagement” Pajulo, 2009 - “In empirical studies of mother-child interaction, substance-abusing mothers have been found to be…less emotionally engaged” Pajulo 2006
(2) **Communication**
"Substance abusing mothers coupled with substance-exposed infants, all wielding their individual difficulties, are at exceptionally high risk for impaired communication patterns” “Communication failure is the result of the difficulty of the mother in reading and interpreting infant cues, and the inherent difficulty of the substance exposed infant to produce meaningful cues that his or her mother can discern” Jansson, 1999 - “These mothers [with substance use] usually have a reduced capacity to read their child's communicative signals…” Pajulo 2011
(3) **Bonding**
“Most of the opioid-exposed infants suffered from early withdrawal symptoms that may have significantly interfered with early bonding.” Salo 2010 - “Closeness, selflessness, purposeful contact, feeding, and eye contact are critical antecedents for maternal-infant bonding in NOWS dyads. These antecedents are similar to the antecedents of maternal-infant bonding for uncomplicated mother-baby dyads. Yet, they are considerably more challenging to achieve for the opioid-exposed mother and baby.” Sanders 2022
(4) **Attachment**
“A parent's capacity to develop a psychological understanding of their child positively influences behaviour and the quality of their emotional interaction, parental beliefs and internal representation of the child, which provide the foundation for a secure attachment relationship.” Perry 2015
(5) **Responsiveness**
“…the ordinal pattern predicted by this hypothesis…refers to the appropriate responsivity of maternal behavior in dyadic interaction…” Brinker 1994 - “Mother-infant interaction may also be affected by qualities of the mother such as decreased responsivity; a lower quality of physical contact; rejecting, ignoring, neglecting, interfering, and insensitive behaviors; maternal preoccupation; and attention and perception problems.” Britt 1994
(6) **Reciprocity**
“In general, polydrug use in mothers has been found to decrease the likelihood of … dyadic reciprocity during interactions” Salo, 2010 - “[Authors] found drug-using/drug-exposed mother-infant dyads to exhibit deficits in enthusiasm, arousal, and mutual enjoyment. They also expressed concern in terms of the dyads' reciprocity, which fell below the threshold of optimal functioning.” Brinker 1994
(7) **Synchrony**
“The EAS is distinct from other parent-child observational tools in its explicit and central focus on capturing the dyadic, synchronous quality of the interaction.” Goldman Fraser, 2010 - “Assessment tools do not consider dyadic communication and synchrony…” Jansson 2019
(8) **Attunement**
“Emotional Availability (EA) is a relational construct that reflects the overall quality of dyadic attunement between caregiver and child” Goldman Fraser 2010 “Affect attunement is a process in which the parent uses a variety of behaviors to convey to the infant that its affect is shared and understood.” Nardi 2000

## Discussion

A focus on the opioid-exposed dyad is critically important as the wellbeing of both birthing person and infant directly influences one another, including considerations of short and longer-term health outcomes (28). Although the dyad is intuitively understood as important and appears as a term throughout the published literature, the present findings suggest that it is not always defined when used and any descriptions can vary significantly.

Notably of the 110 articles identified with a focus on the opioid-exposed dyad, no articles contained a specific definition, either formal or operative. Although the primary emphasis was on identifying criteria and/or definitions, articles with a referenced description of the dyad were included in this literature review. Unexpectedly, 70% of articles identified with a focus on the dyad did not include a description beyond a reference to the dyad as a birthing person and infant (or related terms, e.g., mother-infant). However, the remaining 30% of articles illustrated key descriptive elements that can be used to characterize the dyad. Synthesis of these findings revealed one concept integral to defining the dyad, the interactional relationship between the birthing person and infant. This is highlighted by integration of the eight key elements identified: engagement, communication, bonding, attachment, (mutual) responsiveness, reciprocity, synchrony, and attunement. Although bonding reflects a directionality in the interaction from birthing person towards infant, it is an essential component in the foundation of this relationship.

These eight elements have been prominently discussed in the literature for decades—spanning maternal-child health, child development, education, and psychology (12, 17–27, 29). The current recommendations represent the first application and integration of these distinct elements to clinically define the opioid-exposed dyad. Notably, they present a concept shift from the opioid-exposed dyad historically representing a linkage between two individual patient populations to an emphasis on their underlying relationship. Such a shift in focus that emphasizes the relationship as definitional for the dyad can not only inform clinical care at the bedside for birthing persons and their infants, but also public health data collection and research aimed to improve short- and long-term outcomes.

Given the perinatal scope of this analysis, the criteria developed here focus on opioid-exposed dyads. However, synthesis of the elements identified during this literature review that support these recommendations suggest applicability to other dyadic populations including non-opioid substance exposures which were included as polysubstances in this analysis. Likewise, these recommendations could be applicable beyond infancy for prenatally opioid-exposed children, which were also included in this study.

Application of these recommendations to non-opioid/substance exposed populations merits further consideration. Although the recommendations were derived from literature on opioid exposure, clinical consideration of the dyad as a relationship prioritizes the interaction between birthing person and infant regardless of the presence and/or type of exposures. Thus, it is noteworthy that while developed specifically for opioid-exposed birthing person-infant dyads, these recommendations bear consideration with respect to clinical care for non-exposed dyads. Specific to opioid-exposed populations, it is important to better understand how the exposure affects the interactional dynamic between birthing persons and their infants. Emphasis on addressing this question—while considering the distinct clinical and social needs of birthing persons and infants—is fundamental to the development of a dyadic-centered approach to care.

Recent recommendations for dyadic-centered care for opioid-exposed birthing persons and their infants highlight the need to establish how the dyad is defined at the bedside (7–10). Although the concept of the dyad has typically indicated a linkage of two individual patient populations, a better understanding of what this constitutes from a clinical perspective remains limited. The absence of more definitive data has contributed to variability in the development and implementation of a dyadic care approach, with downstream effects on the birthing person and infant, public health data collection, and research efforts. A shift in focus to the dyad as a relationship as presented here can inform a dyadic care approach across these domains: (1) clinical practice; with an emphasis on understanding clinical and social needs specific to improvement of the interactional dynamic between birthing person and infant (e.g., to tailor supportive service linkage to address specific wraparound needs) as opposed to addressing the needs of one or the other aspect of the dyad (e.g., providing simultaneous care to the dyad, addressing the health care needs of each, while contemporaneously evaluating and assisting the interactional capacities of the pair). Such a model could be cost effective—with one healthcare setting for both—and provide superior care in the short and long term, as dyadic interaction/communication is related to child development and maternal parenting capacities for the child, (2) surveillance; with an emphasis on public health issues pertinent to supporting the dyadic relationship through data collection that informs health and supportive programs (e.g., standardization of protocols, metrics specific to public health follow-up, the linkage of child and parent in the derivation of public health policy), and (3) research; with an emphasis on better understanding the dynamics of the dyadic relationship through clinical studies with consistency in inclusion criteria and outcome measures (e.g., short- and long-term outcomes for the dyad as opposed to the individual components). Altogether, a consistent definition addresses a key knowledge gap in the development of dyadic-centered care.

This literature review is subject to limitations inherent of a non-systematic review. While an internal fidelity process was developed to minimize inter-reviewer differences, introduction of reviewer bias may have influenced the findings. The keyword search was informed by an environmental scan and developed using a comprehensive approach applying both historical and new terminology in the field. Nonetheless it may have missed articles for inclusion. Additionally, the literature review was specifically designed with a focus on the perinatal period, by concentrating on articles inclusive of the birthing person and infant relationship (birth to age one year). While it is conceivable that a similar dyadic relationship could be applicable to older children who had been exposed to parental opioid/substance use during pregnancy, current recommendations are specific to infants as is consistent within the scope of the infant-specific dyadic characteristics (e.g., bonding, attachment) identified from this literature review. It is also possible that beyond the birthing person-infant dyad, a focus on this interactional relationship is one that can be applied to infants with other primary caregivers such as family members and foster or adoptive parents. However, given the study's scope on prenatal exposures, this conclusion is not one that can be made from these findings, but one that merits future research.

## Conclusions

This literature review identified eight descriptive elements characteristic of the birthing person-infant dyad: engagement, communication bonding, attachment, responsiveness, responsiveness, synchrony, attunement. By integrating these elements, a clinical definition of the dyad was developed based on one fundamental principle, the interactional relationship between the birthing person and infant. This definition shifts the concept of the opioid-exposed dyad from a linkage between two individual patient populations to an emphasis on their underlying relationship. Created for clinical use, this definition can inform development of a dyadic-centered approach to care with downstream public health data and research considerations for this vulnerable population.

## Data Availability

The original contributions presented in the study are included in the article/Supplementary Material, further inquiries can be directed to the corresponding author.
