# Supplementary material for: Clinically defining the opioid-exposed birthing person and infant as a dyad to support bedside care, surveillance, and research
Source: Front Pediatr. 2024 May 7;12:1349102. doi: 10.3389/fped.2024.1349102 (PMC11106404; doi:10.3389/fped.2024.1349102)
Supplement: Supplementary file 1 [file Table1.pdf]

Supplementary Table 1. Full-Text Articles with a Description Referenced for the Birthing Person-Infant Dyad. Articles which included a description for use of the terms dyad and/or pair (n = 33) organized by: 3 topical categories, study focus, descriptive reference to the dyad abstracted from a structured full-text search, and descriptive elements identified from thematic analysis.

**Topic Group 1: Types of Birthing Person-Infant Support and Outcomes (n=21)**				
**Year**	Author	Focus	Descriptive Reference to the Dyad	Descriptive Element
**2022**	Sanders	To use Rodgers' evolutionary concept analysis in a literature review to explore the concept of maternal-infant bonding for the opioid-exposed mother-infant dyad	-"Closeness, selflessness, purposeful contact, feeding, and eye contact are critical antecedents for maternal-infant bonding in NOWS dyads. These antecedents are similar to the antecedents of maternal-infant bonding for uncomplicated mother-baby dyads. Yet, they are considerably more challenging to achieve for the opioid-exposed mother and baby."<br>-"Social support and high self-esteem are two factors that positively impact maternal-infant bonding for opioid-exposed mother-infant dyads."<br>-"Second, while maternal infant bonding for dyads not exposed to opioids was most often described in terms of the maternal feelings and emotions about her pregnancy and newborn, the concept of bonding for the opioid exposed dyad may be even better defined by clinicians and researchers who included behavioral and lifestyle components in their definitions."	- Bonding<br>- Attachment
**2022**	Sarfi	To describe parenting stress, perceived child behavioral problems, and postnatal mental health of mothers in opioid maintenance therapy and a comparison group of nondependent mothers, and to explore the association between child behavior problems and parenting stress in the two groups	-"Studies of early mother-child interaction have consistently found patterns of poor sensitivity and responsiveness to infants' emotional and behavioral cues in dyads of substance-dependent mothers compared to normative dyads."	- Responsiveness<br>-

**2021**	Reese	To provide an integrated mechanistic model of perinatal opioid use, parenting behavior, infant attachment, and child well-being to inform behavioral interventions for opioid-exposed mother–infant dyads	-"Decades of animal and human research on parenting has revealed an "intricate interplay of numerous neural, mental, and behavioral processes of perception, motivation, affect, cognition, … and motor performance in shaping the mother's behavior to engage in a selective and enduring reciprocal emotional relationship with [her child]." This dynamic, interactive process occurs within the dyad, with reciprocal influences between infants and mothers."<br>-"This dynamic dyadic system is known as emotion co-regulation and is operationalized as the dyad's shared gaze, complementary affective states, verbal turn-taking, and interactive behavior. Dyadic synchrony can help a child learn to self-regulate through the mechanisms described above."	- Reciprocity<br>- Synchrony
**2017**	Hatzis	To assess the extent to which mothers with substance misuse have compromised caregiving by providing a comprehensive systematic review and meta-analysis of studies that have compared the quality of parent-child interactions in illicit substance-misusing and non-misusing groups	-"The compromised caregiving seen in mother-infant dyads has been implicated in poor outcome and high rates of disorganised and insecure attachment."<br>-"The primary outcome measure was maternal sensitivity. This was operationalised as a maternal response to infant or child cues related to maternal warmth in situations of low frustration rather than during situations of frustration or negative affect. The secondary outcome measure was child responsiveness…and 'child responsiveness indicates how well infant responds to maternal bids and expressions.'"	- Attachment<br>- Responsiveness
**2016**	Maguire	To describe the interactions between mothers in a methadone treatment program and their infants during a bottle feeding and compare the findings with normed data, using a comparative-descriptive design	-"attachment serves as the foundation of healthy functioning in childhood and adulthood." Both members of the dyad, a disorganized infant with NAS and a mother with a substance use history, interact in complex and observable ways to play a part in the creation of each child's attachment."<br>-"A decline in attunement to infant cues through maternal addiction has been seen in neuroimaging and dyadic observation."<br>-"A wide variety of early interventions to enhance the pleasure of parenting and dyadic attunement have shown a change in	- Attachment<br>- Attunement

			maternal sensitivity and in physiologic regulation, trust, and attachment outcomes in children"<br>-"This model illustrates how effective mothers continually use previous infant cues and responses to adapt to more effective parenting behaviors and therefore more successful interactions between the mother and baby."	
**2016**	Porreca	To longitudinally monitor parental abilities in four women with substance addiction who presented four distinct patterns of adult attachment representations, aimed to investigate whether the development of dyadic patterns of interaction with their children varied	-"Drug addiction is considered a major risk factor that can influence maternal functioning at multiple levels, leading to less optimal parental qualities and less positive interactive exchanges in mother-child dyads."<br>"More specifically, we aimed to investigate whether the development of dyadic patterns of interaction with their children varied on the basis of maternal attachment representations."<br>-"As an expansion upon the original conceptualization of the parent-child attachment relationship, emotional availability appears a particularly useful concept in this sense; in fact, it recalls in part sensitivity as referred to by attachment theorists but at the same time adopts a wider perspective, emphasizing the "emotional features" of adult child-interactions, intended both as the ability of the parent to adequately signal and to correctly perceive infant emotional bids. Beyond sensitivity, emotional availability focuses on other aspects of adult behaviors (structuring, non-intrusiveness and non-hostility) and gives equal importance to the child's contribution, considering his/her ability to respond to the parent and to appropriately involve him/her during interactions"	- Attachment
**2015**	Newman	To implement a rooming-in program to support close uninterrupted contact between opioid-dependent women and their infants, using a cohort study approach	-"Separation of mother-infant dyads in the early postpartum period is detrimental to the development of mother-infant bonding and attachment."	- Bonding<br>- Attachment

**2012**	Belt – Infant Mental Health Journal	To examine the outcome of psychodynamic mother–infant group psychotherapy outpatient intervention or psychosocial support for substance-exposed mother-infant dyads compared to a non-drug-abusing comparison group	-"Dyadic responsiveness was operationalized as mutual attention, positive affects, turn-taking, maternal pauses, infant clarity of cues, and maternal sensitivity"<br>-"Psychosocial Support (PSS) Intervention started perinatally, focusing on the dyadic mother–infant relationship to enhance maternal well-being and to prevent disturbances in child development."<br>-"We hypothesized that both interventions would improve the quality of dyadic interaction in maternal sensitivity, structuring, nonhostility, and nonintrusiveness as well as in child involvement and responsiveness"<br>-"However, we expected more positive changes in psychoanalytic mother-infant therapy groups (PGT) than in PSS mother–infant dyads due to its focus on supporting mothers in the peer group to regulate their own emotions and to learn dyadic emotion recognition and regulation.<br>-"Furthermore, continuing the mother–infant intervention into the second half-year of the infant's life may essentially help the formation of dyadic attachment relationship…"	- Attachment<br>- Responsiveness
**2012**	Pajulo – Infant Mental Health Journal	To explore maternal pre- and postnatal reflective functioning and its association with background factors, trauma, psychiatric symptoms, postnatal interaction, child development, and later child foster care placement, focusing on a high-risk group of substance-abusing mother-baby pairs in a residential treatment program	-"The negative experience of misunderstanding in dyadic interaction invariably leads to increased risk for child neglect and abuse."<br>-"The [residential parenting] program has been designed to support the mother in her efforts toward abstinence as well as her relationship with the baby during pre- and perinatal periods."<br>-"The capacity for reflective functioning (RF) is considered a prerequisite for parental sensitivity in parent-child relationships. RF also is considered a mediator in the intergenerational transmission of attachment security, and plays a critical role in shaping maternal representations of children that are characterized by understanding and caring versus indifference and rigidity." [RF refers to the psychological processes underlying one's capacity to understand oneself and others in terms of mental states and to reason about one's own and others' behavior in relation to mental states."]	- Attachment

			-"Together with mental health problems and other psychosocial vulnerabilities, the neurological and developmental deficits associated with maternal addiction are easily transmitted during mother-baby interactions."	
**2011**	Pajulo – Nordic Journal of Psychiatry	To explore amount and type of maternal postnatal psychiatric symptoms, relationship with the baby, and their association with later need for child substitution care placements among mother-baby pairs in a residential treatment program	-" these mothers [with substance use] usually have a reduced capacity to read their child 's communicative signals, and the negative experiences of dyadic interaction lead to increased risk for child neglect and abuse."<br>-" The Care Index for infants and toddlers is an assessment of playful interaction occurring under nonthreatening conditions"<br>-"Three aspects of maternal interaction are assessed: sensitivity, controlling behavior and unresponsive behavior. Sensitivity refers hereby to mother' s ability to notice, interpret and react to infant signals, accurately enough and timely enough."	- Communication
**2010**	Goldman Fraser	To investigate emotional availability among mothers benefiting from participation in a substance-abuse treatment program using an exploratory research design	-"Emotional Availability (EA) is a relational construct that reflects the overall quality of dyadic attunement between caregiver and child. A growing collection of research has directly investigated EA using the *Emotional Availability Scales"*<br>-"The EAS is distinct from other parent-child observational tools in its explicit and central focus on capturing the dyadic, synchronous quality of the interaction."<br>-"The EAS measures several dimensions of the dyadic interaction: parental sensitivity; parental controlling behavior in the form of effective structuring and nonintrusiveness; hostility; child responsiveness; and child involvement."	- Responsiveness<br>- Attunement<br>- Synchrony
**2010**	Salo	To examine cognitive development and mother-infant interaction among prenatally opioid-exposed infants whose mothers participated in drug-replacement therapy vs. unexposed dyads	-"In general, polydrug use in mothers has been found to decrease the likelihood of maternal contingent responsiveness and dyadic reciprocity during interactions"<br>-"Emotional availability (EA) was assessed based on 4-min videotaped observations of mother–infant freeplay, using the EA Scales." "The EA Scales include four adult scales (sensitivity, structuring, nonintrusivenes, and nonhostility) and two child scales (responsiveness to the adult and involvement of the adult).	- Bonding<br>- Responsiveness<br>- Reciprocity

			-"However, since interaction is bidirectional, the low level of maternal emotional availability may also be, in part, child-led. Most of the opioid-exposed infants suffered from early withdrawal symptoms that may have significantly interfered with early bonding."	
**2009**	Pajulo – J Prenat Perinat Psychol Health	To explore factors related to treatment outcome, including quality of mother-child interaction, abstinence from substances and child development, of an ongoing residential intervention using an observational, prospective follow-up design	-"Sensitivity to infant signals is considered as a dyadic construct; as any pattern of adult behavior that pleases the infant and increases the infant's comfort and attentiveness and reduces its distress and disengagement."<br>-"[Substance-exposed] mother-baby pairs have shown fewer moments of dyadic interaction, the quality of the dyadic interaction has lacked enthusiasm and mutual enjoyment, and has included more dyadic conflict and less mutual arousal"	- Engagement
**2006**	Pajulo – Infant Mental Health Journal	To summarize the specific aspects for consideration and previous research findings regarding residential treatment of mothers and their children	-"In empirical studies of mother-child interaction, substance-abusing mothers have been found to be less sensitive in interaction with their children, less emotionally engaged, less attentive, resourceful, flexible and contingent, toexperience less pleasure in the interaction and to be more intrusive in their behavior. Substance-exposed children have been found to show less positive emotion during the interaction, more distress to novelty, a slower recovery from interruptions, an impaired response to stress, and a diminished ability to persist in a task or maintain an alert, attentive state. The pair shows fewer moments of dyadic interaction, the quality of the dyadic interaction lacks enthusiasm and mutual enjoyment, and includes more dyadic conflict and less mutual arousal."<br>-"When maternal reflective abilities are well developed, children are more prosocial, responsive, better able to regulate their emotional state, and the dyadic relationship is more congruent"	- Engagement
**2005**	Savonlahti	To explore the early interaction capacities among high-risk, substance-dependent	-"The Parent-Child Early Relational Assessment (P-CERA) has been developed to assess the areas of strength and concern in the parent, child, and dyadic interaction. The behavior, affective state and communicative skills of the parent and child are	- Communication<br>- Reciprocity

		mother-infant pairs in residential treatment with a comparison low-risk group of mother-infant pairs	assessed. The dyad's affective quality and mutuality are also evaluated."<br>-"Infant and dyad items are used in this study. The infant items (25) are divided into four subscales, and the dyadic items (8) into two subscales, which are Affective Quality of Interaction (Anger, Hostility, Irritability; Flat, Empty, Constricted; Tension, Anxiety; Enthusiasm, Joyfulness, Mutual Enjoyment, A Sense of Dyadic "Joie de Vivre") and Mutuality (Joint Attention, Activity; Reciprocity; Organization/Regulation of Interactions; State Similarity)."<br>-"The total number of concern scores for dyad interactive evaluation, indicating a deficiency in dyadic interactive capacity, ranged from 0 to 8 in feeding and free play situations separately, and from 0 to 16 in both situations together."<br>-"Mutuality is one of the most significant psychological contexts, in which the infant learns to improve self-regulation capacities through dyadic regulation, to actively participate in her/his own turn to interact and to react to mother's initiations and get responses to her/his signals."	
**2000**	Nardi	To describe the development and testing of an instrument that can be used to assess the developing relationship between parents and their young children, during drug or<br>alcohol addiction recovery used for dyads receiving outpatient addiction treatment	-"Attachment is a process of interaction between two partners in the parent-child relationship that evolves over time from a need of the infant to be physically safe and connected to the caregiver to the more complex human need to be emotionally safe and socially connected to others."<br>-Affect attunement is a process in which the parent uses a variety of behaviors to convey to the infant that its affect is shared and understood. This communicating of one's understanding of the nature of another's experience, or empathy, is a critical component of parenting. It allows the parent to enter the infant's world and initiate the mutuality that is the cornerstone of the attachment relationship."<br>"Eighteen mother-child dyads, consisting of a mother admitted for outpatient addiction treatment and her child, were assessed each week by the primary therapist using the PTF [Parenting Tracking Form]. The PTF was developed to examine the parent-child relationship in an outpatient perinatal addiction program."	- Engagement<br>- Communication<br>- Attachment<br>- Responsiveness<br>- Reciprocity<br>- Attunement

			-"PTF Scale Mother-child interaction: "Mutual engagement: In terms of mutually engaging in activities or events at the program site or in the nursery, maternal-child dyads characteristically engaged in joint attention and activity; Reciprocity, Turn-Taking: Contingent responsivity and engagement on the part of both partners in the maternal-child dyad; Mutual enjoyment: A joyful, enthusiastic pairing and a sense of dyadic joie de vivre"	
**1999**	Jansson – Inf Young Children	To describe the multiple needs of the substance abusing mother and infant and the experience of a comprehensive care treatment facility in meeting those needs	-"Substance abusing mothers coupled with substance-exposed infants, all wielding their individual difficulties, are at exceptionally high risk for impaired communication patterns, poor mother-infant bond, and child neglect and abuse."<br>-"Communication failure is the result of the difficulty of the mother in reading and interpreting infant cues, and the inherent difficulty of the substance exposed infant to produce meaningful cues that his or her mother can discern."	- Communication<br>- Bonding
**1997**	Burns	To observe the details of the mother-infant relationship of dyads with and without maternal substance use, and to identify the factors which differentiate the two groups	-"If either mother or infant begin this relationship with significant skill deficits which interfere with their ability to relate, such deficits may place the dyadic relationship at risk for long term dysfunction."<br>-"The most striking deficit in dyadic functioning found by these researchers was the lack of enthusiasm and mutual enjoyment of these mothers and infant for one another."<br>-"*The Parent-Child Early Relational Assessment* which was developed to assess maternal behavior from the point of view of the infant, and to assess infant behaviors from the point of view of the mother. The ERA measures maternal affect, parental style and mood, attitude toward the child and behavioral involvement with the child. It also includes items rating the infant's mood, affect and behaviors, as well as items characterizing the quality of the interaction and mutuality of the dyad."	- Responsiveness<br>- Reciprocity
**1994**	Brinker	To determine the extent to which four hypotheses about differences in mother-child interaction patterns are	-"The first hypothesis, the natural intersubjectivity hypothesis, is a specific variant of the notion that there is a primary intersubjectivity that is part of species specific human maternal behavior. Thus, the ordinal pattern predicted by this hypothesis…refers to the appropriate responsivity of maternal	- Responsiveness<br>- Reciprocity

		associated with the family socioeconomic status, the characteristics of infants, or special problems such as in utero drug exposure and ongoing maternal drug usage for dyads enrolled in a childhood intervention program	behavior in dyadic interaction and the numerals refer to sequential points in time."<br>-"[Authors] found drug-using/drug-exposed mother-infant dyads to exhibit deficits in enthusiasm, arousal, and mutual enjoyment. They also expressed concern in terms of the dyads' reciprocity, which fell below the threshold of optimal functioning."<br>-"the Parent-Infant Interaction Scale and the Teaching Skills Inventory…both scales provide macro analytic ratings of the responsivity and sensitivity involved in the dyad's interaction as well as the roles played by both participants."	
**1994**	Britt	To test the effectiveness of the Neonatal Behavioral Assessment Scale and the Mothers Assessment of the Behavior of the Infant as interventions to enhance mother-infant interaction with low-income, drug using mothers, by randomly assigning dyads to 1 of 4 interventions	-"Mother-infant interaction may also be affected by qualities of the mother such as decreased responsivity; a lower quality of physical contact; rejecting, ignoring, neglecting, interfering, and insensitive behaviors; maternal preoccupation; and attention and perception problems."<br>-"Mother-infant interaction, a variable used to evaluate the level of intervention effectiveness, was assessed via the Nursing Child Assessment Feeding Scale. This was chosen as an outcome measure because it is a standardized measure of mother-infant interaction. Maternal subscales include sensitivity to infant's cues, responsiveness to distress, social-emotional growth fostering, and cognitive growth fostering. Infant subscales include clarity of cues and responsiveness to parent."	- Responsiveness
**1982**	Johnson – Infant Mental Health Journal	To develop a method to assess high risk families in the newborn period based upon data collected for mother-infant pairs using a screener tool, and describing its scoring and interpretation	-"the screener [called Borgess Interaction Assessment – BIA] includes items which assess factors in the parent's past, the mother's intrapartum responsiveness and attitudes to the infant and her interactions with the baby during the hospital. The screener is filled out by nurses providing care to the mother and infant."<br>-"The purpose of the BIA is to assess the number and severity of conditions/situations/experiences which pose a threat to attachment and positive interactions"<br>-"The BIA…includes twelve items which assess the following conditions: infant's vulnerability, experiences of the mother which may impair attachment, potential stressors which could impair nurturance."	- Attachment<br>- Responsiveness

Topic Group 2: Presence or Types of Opioid/Substance Exposure and Outcomes and/or Care Recommendations (n=7)				
**Year**	Author	Focus	Descriptive Reference to the Dyad	Descriptive Element
**2020**	Beauchamp	To investigate the extent to which prenatal substance exposure impacts infant self-regulation during a relational stressor and the association between self-regulation and infant affect with exposed and non-exposed dyads	-"The Still Face Paradigm involves a series of interaction episodes between an infant and a caregiver in which the caregiver shifts from interacting with to ignoring the infant, which is experienced by the infant as a stressor. A modified version of the original SFP was used that included a total of five episodes, each 120 s in length: Episode 1) a baseline play episode to determine typical maternal-child interaction patterns for the dyad"	-Responsiveness
**2019**	Jansson – Pediatr Clin North Am	To examine the continuum of care of opioid-exposed infants, including the assessment of the neonate, diagnosis and management of neonatal abstinence syndrome, pediatric follow-up care, and integration of care of the mother-infant dyad	-"assessment tools do not consider dyadic communication and synchrony (ie, the mother's ability to read, interpret, and respond appropriately to infant cues and the ability of the infant to effectively relay needs to the mother"<br>–"Nonpharmacologic care of the maternal-infant dyad affected by neonatal abstinence syndrome. Assessment Functioning of the Dyad; With the Goal of Bidirectional communication and dyadic synchrony"	- Communication<br>- Synchrony
**2016**	Savin	To provide a review of clinical and legal considerations for opioid exposed mothers and infants in Delaware	-"Due to increased numbers of opiate exposed mother-infant dyads, the stress and challenge of caring for vulnerable families is only increasing. The caregiver, mother, and infant are in a continual feedback loop with the actions of each affecting the reactions of the other."	- Reciprocity
**2015**	Jansson – J Perinat Neonat Nurs	To identify barriers to lactation in substance-exposed dyads and provide strategies to mitigate these barriers and to promoting lactation	-"Substance-exposed infants may be unable to transmit easily interpretable cues that can affect dyadic communication."<br>"As important as it is to understand the infant and the mother, it is equally necessary to understand the dyad and their communication, such as the substance affected infant's capacity to transmit interpretable cues (ie, indicating hunger,	- Communication<br>- Responsiveness

			overstimulation, the need for position change) and the maternal interpretation and ability to provide a sensitive response to those cues."	
**2015**	Perry	To assess psychological and psycho-social risk factors for substance dependent women exposed to high risks in pregnancy, and their impact on child protection involvement with a group of pregnant women on opioid substitution treatment vs. pregnant women without substance use	-"A parent's capacity to develop a psychological understanding of their child positively influences behaviour and the quality of their emotional interaction, parental beliefs and internal representation of the child, which provide the foundation for a secure attachment relationship."<br>-"*Emotional Availability Scales:* This instrument is designed to evaluate the quality of communication, connection and emotional tone between an adult and a child. The Emotional Availability scale was used to assess the quality of mother–child connection during a 15-minute unstructured videotaped dyadic interaction between mother and infant. Emotional availability was categorised into four adult dimensions – the parent's sensitivity; structuring; nonintrusiveness and non-hostility toward the infant; and two child dimensions – responsiveness and involvement toward the adult. "	- Communication<br>- Attachment<br>- Responsiveness
**2008**	Velez – J Addict Medicine	To review the contribution of maternal opioid dependency to the difficulties experienced by the mother-infant dyad and their treatment providers in the postnatal period, the nonpharmacologic treatment of the infants, with emphasis on the treatment of the mother and baby as an interactional dyad	-"The discussions and recommendations provided will assist health care providers with viewing the opioid exposed infant as one aspect of a dyad and the need for simultaneous treatment of both the infant and the mother."<br>-"Attention to maternal reactions and behaviors can direct the intervention with the dyad to diminish emotional overload and provide external organization until the infant can develop higher sensory limits and consistent behavioral self-regulation. Helping the mother to be aware of her emotions, behaviors driven by those emotions, and her capacity to regulate them may improve her ability to respond to the infant and thereby the mother infant interaction."	- Responsiveness
**1990**	Johnson – Amer J Ortho-psychiatr	To examine behavioral characteristics of a population of mothers at multiple risk, including	-"The importance of mother-infant interaction to the infant's emotional and cognitive development has been demonstrated repeatedly."	- Engagement

		drug use during pregnancy, and their infants using videotaped mother-infant interactions	-"Each videotape of mother-infant interaction at two, four, and six months was coded: activity, adaptability, approach, intensity, mood, distractibility, and persistence. Maternal behavior was coded on five dimensions- interacting, negative participation, apathy, vocalization, and holding. Interacting was defined as behavior in which the mother makes an active effort to engage the child, i.e., her behavior seeks and encourages responses."	
**Topic Group 3: Psychiatric Illness, Opioid/Substance Use/Exposure, and Outcomes (n=5)**				
**Year**	Author	Focus	Descriptive Reference to the Dyad	Descriptive Element
**2014**	Siqveland – Infant Behavior & Development	To investigate the development of mother-infant interaction patterns among three groups of mother-infant pairs recruited during pregnancy: from residential substance abuse treatment, psychiatric outpatient treatment and well-baby clinics	"The adversities facing both the mother with substance abuse problems and her infant might impact negatively on the mother–infant interaction, and lack of reciprocal enjoyment and enthusiasm in the mother–infant interaction has for instance been found among such dyads."<br>-"The mother–infant interaction at 3 and 12 months was assessed by the Parent–Child Early Relational Assessment (PCERA), which is a video-based, parent–child assessment method that aims at capturing the strengths and areas of concern in the parent, the child and the dyad. The two dyadic subscales include "affective quality of interaction" (4 items) and "mutuality" (4 items)."<br>-"Dyadic mutuality in the interaction has been suggested to serve as an important context for the development of infant self-regulation accomplished by infant participation in the back and forth interaction with the mother as well as maternal responses to infant signals"<br>-"Overall the results from this study demonstrate that the dyads with substance abuse problems displayed more relational disturbances in the mother–infant interaction."<br>-" It was further illustrated that both the mother and her infant contributed to the dyadic affective reciprocity, underscoring in line with the transactional model the value of examining both maternal and infant behavior, as part of a dyadic unit."	- Responsiveness<br>- Reciprocity<br>-
**2014**	Siqveland – Child	To investigate the longitudinal development of the affective quality of	-"Maternal substance abuse problems have been associated with a reduced ability to accurately perceive and respond	- Reciprocity

	Psychiatry Hum Dev	the mother-infant interaction during the first year in relation to maternal optimality in four different domains (substance abuse, psychiatric problems, relational experiences, socioeconomic status) comparing mothers enrolled during pregnancy from substance abuse treatment institutions, a psychiatric outpatient center and well-baby clinics	appropriately to infant emotional distress and with deficits in dyadic reciprocity in mother–infant interactions."<br>-"The mother–infant interaction at 3 and 12 months was assessed by the PCERA. The two dyadic subscales (8 items) include Affective quality of interaction and Mutuality. The subscale affective quality of interaction, comprises four items, including anger and hostility, flat and empty, constricted, tension and anxiety. It also measures the enthusiasm, joyfulness, and mutual enjoyment in the dyad."<br>-" Papousek suggests a model of infant–parent emotional regulation and communication in which two types of dyadic circles are described. A positive reciprocity between the mother and infant will promote a so-called ‘‘angels circle’’ of co-regulation, while negative reciprocity in the mother–infant interaction will result in a vicious circle of dysregulation."<br>-"Results from the present study indicate that long-term interventions may be needed to promote the affective quality of the interaction among mother-baby pairs affected by substance abuse and other co-existing non-optimal factors, in order to prevent an attenuation of maternal emotional involvement and dyadic reciprocity over time."	
**2012**	Siqveland – Infant Behavior & Develop-ment	To investigate the predictive validity of four different optimality indexes and infant perinatal status in relation to maternal sensitivity in interaction comparing mothers with substance abuse and psychiatric problems who underwent treatment during pregnancy and mothers without substance abuse or psychiatric problems	-"mother–infant interaction is dyadic and bidirectional. It involves both the mother's ability to interpret and respond to signals from the baby, and the infant's own capacity to modulate his states and be able to signal and respond to his caregiver."<br>-"The PCERA consists of 65 variables that are clustered into different parent, child and dyadic scales, respectively. The scale consists of several items including the quality and amount of physical contact, visual contact and verbalization. It also measures the caregiver's social initiative, actions to structure and mediate the environment, ability to read the child's cues and respond appropriately, level of connectedness and mirroring, and contingent responsivity to the child's positive or age-appropriate behavior."	- Responsiveness

**2011**	Sidor	To replicate previous research: that the severity of maternal depression is inversely related to maternal sensitivity in a dyadic interaction – for mother-infant dyads at psychosocial risk – with an intervention and control group	-"Based on the current literature, we assumed that the severity of maternal depression would be inversely related to maternal sensitivity in a dyadic interaction."<br>-"The CARE-Index is a dyadic procedure which assesses adult sensitivity in a dyadic context. Each aspect of behaviour is evaluated separately for adults and infants. For adults these are “sensitivity”, “control” and “unresponsiveness”. The infants' scales are “cooperativeness”, “compulsiveness”, “difficultness” and “passivity.”	- Responsiveness
**1996**	Cassidy	To assess adolescent maternal psychopathology – psychosocial adversity, alcohol, or substance dependence – and its relations to the quality of mother-infant interactions with mother-infant pairs recruited from an adolescent medicine-specialized obstetric clinic	-"The CARE index assesses patterns of mother-infant interactions on 3 dimensional categories: sensitivity, controlling, and unresponsive. While separate scores are given to mother and infant, the ratings are ascribed in the context of the other's behaviour. Mothers are rated in 7 different areas (facial expression, vocal expression, position and body contact, expression of affection, pacing of turns, control, and choice of activity). Similarly, the infant interactions are rated on 4 dimensions, cooperative, difficult, compulsive-compliant, and passive, with a score of 14 on the cooperative measure being optimal. While the infant and mother ratings are mathematically independent, previous work has found strong correlations between the following mother and infant dimensions: sensitivity and cooperation; controlling and difficult; controlling and compulsive compliant; and unresponsive and passive”	- Responsiveness
